# Patterns and outcomes of health insurance disputes in Saudi Arabia: an analysis of appealed judicial precedents

**DOI:** 10.3389/fpubh.2026.1908431

**Published:** 2026-10-19

**Authors:** Shahad Alotaibi, Ghufran Alhawdar, Sama’a Almubarak

**Affiliations:** College of Public Health, Imam Abdulrahman Bin Faisal University, Dammam, Saudi Arabia

**Keywords:** health insurance, health litigation, insurance disputes, judicial precedents, Saudi Arabia

## Abstract

**Introduction:**

The expansion of health insurance systems has generated a rise in insurance disputes. Health insurance litigation can have negative consequences spanning beneficiaries, healthcare providers and broader healthcare systems. This dynamic is particularly relevant to Saudi Arabia as its health insurance system is experiencing rapid transformation and growth. Thus, the study analyzed patterns and outcomes related to health insurance disputes in Saudi Arabia.

**Methods:**

The study employed a retrospective analysis of 38 appealed judicial precedents related to health insurance in Saudi Arabia between 2015 and 2024. Data were extracted from published documents by the General Secretariat of the Committees for Resolution of Insurance Disputes and Violations. Extracted data included two dependent variables: final verdict and ratio compensation. The independent variables included variables related to the plaintiff, defendant, insurance policy as well as litigation-related variables. Data were analyzed using descriptive statistics and the associations between the dependent and independent variables were examined using the Fisher’s Exact test.

**Results:**

The largest volume of the judicial precedents originated from the Western region with 16/38 precedents (42.1%). Patients were the primary plaintiffs (55.3%), while insurance companies predominated as defendants (94.7%). The majority of the disputes were related to medical insurance (65.8%), driven primarily by complaints regarding lack of insurance coverage (55.3%). The results indicated that most cases resolved within one year or less. The appeal committee affirmed most verdicts issued by first-instance committees. A total of 26/38 (68.4%) defendants were found liable and 15/38 (39.5%) of plaintiffs were awarded full compensation.

**Discussion:**

The study’s finding reflects a high stability and conformity between committee levels. However, the heavy concentration of cases involving medical coverage denials and patient-led litigation underscores the critical need for clear regulations to minimize insurance conflicts. The findings highlight several implications for policymakers and healthcare practitioners as Saudi Arabia’s health insurance sector continues to expand. Key priorities include enhancing the transparency and systematic publication of judicial precedents, clarifying coverage and exclusion terms in policy contracts, and standardizing the formal documentation of claim denial reasons.

## 1 Introduction

Health insurance serves as a primary healthcare financing mechanism worldwide. With global premiums for medical insurance policies reaching approximately EUR 1.68 trillion in 2024, and policies being implemented in two-thirds of countries, health insurance serves as a massive and critical tool for managing health expenditures (1, 2). By diversifying funding mechanisms, insurance-based financing enhances the sustainability of broader healthcare systems and alleviates pressures on government budgets, shifting a portion of the financial obligation from public expenditure to alternative risk-pooling frameworks (3, 4). While health insurance provides crucial financial protection the long-term sustainability of insurance funds remains a persistent challenge for health systems worldwide. Changing demographics (such as aging populations), escalating service utilization for chronic conditions, and structural healthcare inequalities across regions, continuously heighten operational deficit risks for insurance funds (5). To mitigate these financial strains, recent empirical evidence underscores the importance of administrative oversight. Indicators such as number of discharged patients, average length of stay and pharmaceutical expense ratio, significantly correlate with health insurance overspending risks (6). Such evidence highlights how macro-level demographic pressures and micro-level clinical cost drivers intersect to risk the financial stability of insurance funds.

Despite its utility as a sustainable funding model, the operationalization of health insurance frameworks often reveals significant discrepancies between statutory policy designs and real-world implementation. Indeed, health insurance poses several challenges, such as high insurance premiums and limited scope of coverage for beneficiaries, which may incur high costs for specific services. Moreover, the administrative complexity of health insurance, such as claims processing, contributes to increased healthcare system costs while simultaneously reducing the quality of care provided to patients. Fraudulent providers also pose a challenge to health insurance systems, further complicating procedures, such as billing for services not received by patients, upcoding and unbundling. Such challenges lead to beneficiaries’ dissatisfaction and increased health insurance dispute and thus introduce profound legal complexities (3, 4, 7).

As health insurance systems expand, inherent ambiguities within insurance policies and coverage boundaries have generated a body of literature exploring insurance disputes. Previous empirical studies of health insurance litigation examined the underlying causes of disputes across healthcare systems in Indonesia, Tanzania, Kuwait, and the United States (7–10). These studies have consistently highlighted claim rejections as the main underlying reason for health insurance disputes. Claim rejections can be attributed to several reasons including operational and administrative factors, coding errors, misconduct, and contractual non-coverage reflected in the exclusion of certain services (7, 9, 10). In Kuwait, an analysis of claims within the retirees health insurance program found that uncovered services was the primary reason for claim rejections (10). Furthermore, claim rejections frequently result from operational and administrative factors or discrepancies at the healthcare provider level in the form of insufficient medical documentation (7, 9). In Indonesia, Purba and colleagues investigated causes of claim rejections through a qualitative case study of a health insurance company. Employee interviews revealed that primary reasons for claim rejections originated from healthcare providers, largely due to procedural non-compliance such as incomplete documentation, expired policies, and late claim submissions (9). Other studies have also documented coding errors as another reason for claim rejections. Through a mixed methods study examining rejection patterns related to the National Health Insurance Fund in Tanzania, Fulla et al. (7, 9) reported incomplete codes and mismatches between codes and procedures or price lists. The extant literature also identifies a more severe reason for claim rejections involving intentional billing fraud, such as fraudulent issuance of receipts, alteration of invoices, duplicate submission of claims, and recorded services in bills that were not provided.

Growing empirical evidence indicates that claim rejections patterns are not uniformly distributed, but rather varies by beneficiary socioeconomic statuses. Evidence a United States cohort study utilizing claims data from Symphony Health’s Integrated Dataverse (2017–2020), found that social determinants of health significantly influenced claim rejections. Beneficiaries with lower incomes, Black, Hispanic, and Asian, were more likely to have their claims rejected (8). Similarly, another study in Kuwait found that claims were more likely to be rejected if they involved female beneficiaries under 40 years old, dental or pharmaceutical services, or services provided at higher-level hospitals (10).

A few studies examined health insurance disputes from a legal perspective. Chrizostimo et al. (11) reviewed 14 research papers published between September and October 2017 of lawsuits related to private health insurance in Brazil. The review found that the majority of disputes involved medication (32%) and hospitalization (11%) issues. In another quantitative cross-sectional study, Afonso et al. (12) analyzed 96 lawsuits from a health insurance company’s legal department between 2012 and 2015. Health-insurance related lawsuits were filed mainly due to non-coverage, with the requested services being explicitly omitted from private contracts or unauthorized by regulatory guidelines. In a related study, Sun et al. (13) investigated the mechanisms surrounding fraudulent claims and fund security by analyzing 215 documented cases alongside longitudinal administrative data from China’s medical insurance bureaus (2018–2021). Their findings demonstrate that fraudulent reimbursement practices significantly compromise fund equity and solvency. Such finding was corroborated in a recent study reviewing 30 studies on the economic impact of fraud, waste and abuse in health insurance systems (14). The review demonstrated that such practices impose severe direct and indirect costs that threaten long-term fund sustainability, operational efficiency, and equity.

Moving beyond the analysis of lawsuits, a distinct body of health law research shifts the analytical focus toward finalized judicial precedents to understand long-term regulatory impacts. A few studies have examined judicial precedents in the medical field across different countries including Korea and the United States (15–17). These studies examined judicial precedents in different clinical specialties, ranging from specialized fields of cosmetic surgery (15), to specific procedures such as hernia repair (17) and skin cancer treatment (16). Such studies have documented the increase of medical disputes overtime by reviewing court decisions to determine main patterns of medical disputes (15–17). While these studies analyzing judicial precedents are concentrated in medical specialties, only a single study has extended this analysis of judicial precedents to the domain of health insurance. Wang et al. (18) analyzed 1,547 final verdicts related to coverage denial against private insurance companies from 2018 to 2021 in Brazil. The most common reason for coverage denial was that medications were not included in the Brazilian National Supplementary Health Agency list (43.2%) or were not included in contracts (34.3%). The majority of judicial disputes were favorable to patients, based on precedents from the Supreme Court of Justice (48%), state courts (56%), or the Brazilian Consumer Protection Act (47.7%) (18). While this study investigated how health insurance disputes within the specific Brazilian context, broader empirical evidence remains sparse.

### 1.1 Health insurance in Saudi Arabia (SA)

The establishment of the Council of Health Insurance (CHI) marked the formal regulatory structure for the health insurance system in SA. Founded in 1999, CHI implements, governs, and regulates the cooperative health insurance system under the CHI Law (19). The CHI Law initially mandated medical insurance coverage for non-Saudi employees working in the private sector in 2006 (20). It was then, extended to include all employees working in the private sector and their dependents. Overtime, health insurance coverage was expanded to further mandate coverage for visitors, including Umrah and Hajj pilgrims, in 2020 (21).

In August 2023, the Council of Ministers Decision no. (85) formally established the Insurance Authority as the highest independent entity governing the insurance sector in SA. Its role includes regulating, supervising, and developing the insurance sector, with efforts to enhance market stability, protect beneficiaries’ rights, and ensure fair and transparent insurance practices. While the Insurance Authority is now the overarching regulator for the entire insurance sector, CHI retains its specialized role focusing on health insurance (22).

The General Secretariat of the Committees for Resolution of Insurance Disputes and Violations (hereafter the General Secretariat) is the responsible body for handling disputes and violations related to insurance. Established in 2003, the General Secretariat’s adjudicates legal disputes, coverage contests, regulatory violations, and financial claims between insured individuals, healthcare providers, and insurance companies are adjudicated. The General Secretariat adjudicate these disputes through specialized quasi-judicial bodies: the first-instance committees and an appeal committee.

While regulatory oversight transitioned to the Insurance Authority during the study period, the legal jurisdiction and adjudicative mandate of the General Secretariat remained continuous, ensuring functional continuity in precedent formation and judicial decision-making (23). In 2024, the number of insurance disputes submitted to the General Secretariat reached 25,515 claims. Disputes related to health insurance accounted for 3.21% (*n* = 818) ranking as the second most common type of insurance disputes, after motor insurance (24). This represents a substantial increase in absolute dispute volume over time, surging from *n* = 119 in 2016 to *n* = 818 in 2024 (24, 25).

#### 1.1.1 Health insurance litigation in SA

The literature examining the cooperative health insurance system in SA demonstrates its critical role in enhancing service quality, improving healthcare accessibility, and reducing out-of-pocket expenses (26–28). However, the extant literature simultaneously highlights persistent systemic and experiential challenges across the sector. In a systematic review of 30 studies, Alzahrani et al. (27) confirmed positive structural contributions of cooperative health insurance to the healthcare landscape, yet identified significant barriers including inadequate infrastructure and escalating insurance premiums. These barriers manifest as operational inequities, resulting in disparities between the private and public sectors regarding the speed and equity of healthcare delivery, while financial barriers continue to impact low-income individuals (28).

Moving beyond examining the effectiveness of health insurance in SA, a few studies focused on legal aspects and judicial mechanisms of health insurance disputes in SA. Focusing on medical malpractice insurance, Alkhenizan and Shafiq (29) compared litigation procedures between SA and the United Kingdom. They analyzed decisions of the Saudi Board of Grievances and the British forensic medical committees. The results indicated that the Saudi system was characterized by lengthy litigation procedures, lack of transparency in publishing judgments, and lower compensation amounts compared to the British system. The researchers highlighted the importance of unifying litigation procedures and publishing rulings to increase transparency. Alternatively, Barakat’s (30) study focused on the judicial bodies handling health insurance disputes before and after the establishment of the Saudi Insurance Authority in 2023. It revealed an overlap in jurisdiction between the Saudi Central Bank and the Council of Health Insurance, as each of them had quasi-judicial committees that adjudicate disputes independently. The study recommended the necessity of unifying authority, activating transparency in publishing rulings, and establishing a national database of insurance rulings.

Representing a unique intersection between health insurance and legal perspectives, Alonazi (31) addressed the critical issues of fraud and abuse within the Saudi health insurance system. The study utilized a comprehensive three-tiered analytical approach by analyzing government policies, operational data from 10 insurance companies, and qualitative interviews with stakeholders. The findings revealed that a large percentage of fraud cases occurred within the clinical specialties of dentistry and obstetrics and gynecology, while highlighting low compliance with anti-fraud regulations. The study emphasized the urgent need to enhance coordination among regulatory, supervisory, and judicial bodies to ensure effective enforcement and mitigate persistent ethical and legal risks.

#### 1.1.2 Research gap and significance of the study

The extant literature predominantly focused on analyzing underlying premises behind claim rejections as the primary reason behind insurance litigation or disputes (7–10). The few studies analyzing health insurance disputes focused on the Brazilian health insurance system and focused only on claims related to accessing treatment (12, 18). Thus, a comprehensive examination of litigation within health insurance remains missing. Specific to SA, most previous studies focused on financial, administrative, or organizational aspects of health insurance, while documenting challenges and examining beneficiaries’ satisfaction levels (26–28). Empirical analyses grounded in the specific legal, administrative, and judicial mechanisms of health insurance within SA remain sparse. Thus, scholarly research has yet to examine how challenges manifest as formal legal disputes. This study directly addresses this empirical gap by analyzing judicial precedents and legal dispute resolution mechanisms within SA’s health insurance system. Specifically, it analyzes annual counts of judicial appealed precedents while examining factors related to final verdicts and compensation outcomes.

The practical significance of this study lies in its ability to offer actionable insights for policy makers, judicial authorities, and insurance regulators by identifying structural patterns in dispute litigation, resolving regulatory ambiguities, and establishing empirical evidence to align insurance governance with practical healthcare delivery. The study’s significance is further highlighted by the rapid development and expansion of the Saudi health insurance sector, driven by the Saudi Health Sector Transformation Program, to transition toward universal insurance coverage and increase private sector healthcare participation (32). The influx of beneficiaries and private insurers naturally heightens the volume and legal complexity of contractual disputes. By analyzing judicial health insurance precedents during this critical growth, the study provides various stakeholders with much-needed predictability as the legal system matures. Such understanding offers regulatory bodies and policymakers with concrete data to refine insurance laws and reduce the rate of contested litigation. Identifying how committees adjudicate health insurance disputes provides crucial foresight for insurance companies, healthcare providers, and legal practitioners. This provides more transparent dispute-resolution mechanisms, ensuring that rapid market growth does not compromise equity or patient rights. Reducing judicial ambiguity ultimately lowers systemic financial risks, streamlines operations, and provides a stable economic environment to support the sector’s growth.

The study represents the first systematic analysis of published judicial precedents related to health insurance in SA. Analyzing these precedents is essential because, unlike isolated lawsuits, judicial precedents reveal how courts consistently interpret laws and regulations and thus help in refining them. By converting raw, extensive legal records into structured, empirical evidence, this study establishes a baseline for understanding how health insurance liabilities are treated across different judicial committees. Its value lies in its focus on the most complex and impactful issues in health insurance practices, which enhances the protection of beneficiaries’ rights and contributes to reducing disputes. Ultimately, this creates a vital scholarly benchmark and extends extant literature by examining health insurance litigation, with a focus on analyzing judicial precedents. Such findings allow for global comparative research, understanding the legality of health insurance within the unique context of SA health system and its interplay with the legal system.

## 2 Methodology

### 2.1 Study design

The study used a retrospective design to analyze appealed judicial precedents related to health insurance disputes in SA. It employed systematic content analysis of judicial precedents, a recognized research methodology within empirical legal studies (33, 34). Systematic content analysis converts qualitative text from judicial opinions and administrative rulings into standardized, replicable quantitative data while preserving legal nuance (35).

### 2.2 Data source

Secondary data were extracted from official judicial precedents that are curated and published by the General Secretariat. The General Secretariat defines judicial precedents as guiding references for future review and adjudication, provided they are not superseded by subsequent statutory provisions (Table 1). The General Secretariat publish these precedents to achieve consistency in judgments and unify judicial direction in recurring issues. Judicial precedents are published in the official website of the General Secretariat in the form of reports with the first edition published in 2021, second in 2022 and the last and most recent in December 2025. Each subsequent edition functions as an updated and cumulative corpus, establishing a comprehensive record of appalled judicial precedents related to the insurance sector in SA.

**Table 1:** Operational definitions for study terms.

Terms	Operational definition
Insurance claim	A request submitted to the insurance company by the insured to obtain coverage or compensation based on the insurance policy.
Claim rejection or denial	An insurance company response involving rejection or non-approval of a request for compensation or coverage.
Complaint	An objection submitted by the insured or healthcare provider, or other concerned party (i.e., plaintiff) because of an insurance-related issue.
Formal dispute	A formal conflict or disagreement is filed by the affected party, whether the insured, the service provider, or the insurance company, to be referred to the competent authority for adjudication.
First-instance decision	The first decision issued by the first-instance committee in a filed dispute, reflecting the primary verdict.
Appeal decision	The decision issued by the appeal committee after reviewing the primary verdict, either affirming or reversing it.
Published judicial precedent	A judicial precedent refers to guiding reference or legal ruling that establishes an authoritative principle, serving as a baseline rule to guide judges when deciding subsequent cases with similar facts. In this study, judicial precedents are published by the General Secretariat of the Committees for Resolution of Insurance Disputes and Violations.
Final verdict	A binary outcome variable indicating a final judicial determination by the appeal committee establishing that the respective defendant was deemed liable (guilty) in the in the adjudicated case or not.
Compensation ratio	The awarded financial compensation ratio was calculated by dividing the compensation awarded for the claim by the requested compensation amount and multiplying the result by 100, with outcomes categorized as rejected (0%), partially compensated (>0% to <100%), or fully compensated (100%).

Published judicial precedents undergo a standardized seven-stage selection and editorial workflow prior to release. The workflow involves: (1) exhaustive collection and thematic classification of all appeal committee decisions by policy type; (2) substantive legal screening to exclude redundant cases or those rendered obsolete by subsequent regulations; (3) consolidation of recurring rulings into standardized, generalizable precedent templates; (4) internal legal review of framing and statutory alignment; (5) formal review and official approval by the appeal committee; (6) final report curation, including anonymization of party identities and verification of statutory citations; and (7) indexing, digital publication, and public dissemination. Consequently, the published corpus represents a curated subset of authoritative, legally significant appealed decisions rather than an automatic census of all filed disputes. These judicial decisions are considered reliable, verified, and publicly available (36–38).

### 2.3 Data collection

Data were manually extracted from the judicial precedent texts using a standardized codebook. Since published precedents include all precedents related to the insurance sector, the study only included judicial precedents related to health insurance. Across the three cumulative archive editions, 96 judicial precedents related to health insurance were identified. After screening, 58 duplicate precedents were identified and excluded. A total of 38 unique judicial precedents comprised all publicly released appealed precedents within the health insurance sector and represented the study’s dataset.

Each judicial precedent was reviewed individually and manually to identify and extract the relevant variables based on the objectives of the study. To ensure inter-rater reliability and minimize subjective interpretation bias, two researchers independently extracted data from the full dataset (*n* = 38). Following independent coding, the two extractions were systematically compared. Any initial discrepancies or ambiguous interpretations were referred to a third senior researcher for formal review. All discrepancies were thoroughly evaluated and resolved through joint review and consensus with the third researcher, yielding the finalized dataset.

The published judicial precedents were anonymized and included only general demographic information such as nationality and gender. Each judicial precedent contained important information for the study such as parties involved, detailed explanation of the cases from case filing until the final judgment along with verdicts of first-instance committees and the appeal committee, compensation amounts, and final verdicts. The 38 published judicial precedent records encompassed a broad spectrum of civil and administrative health insurance disputes related to medical insurance and medical malpractice polices. Eligible matters included contractual coverage denials, lack of patient insurance coverage, policy condition violations, insurance premium calculations, provider fee-for-service claims, and medical malpractice professional liability indemnity. The number of pages for each case ranged from three to 10 pages, with detailed information, and written in formal Arabic language.

Extracted data were then entered into a developed data collection tool. For each judicial precedent extracted data included the dependent variables: final verdict and ratio compensation. The independent variables included geographical location, medical specialty, plaintiff, plaintiff gender, plaintiff nationality, defendant, duration from case filing to final verdict, type of case, precedent classification, primary verdict, and appeal committee decision. The variable geographical location referred to the location where the committee hearing was held. Cases in which the hearing location was not reported were coded as missing (NA). The variable clinical specialties reflected the respective specialty of each analyzed precedent. Specialties with low individual representations, specifically speech and occupational therapy (*n* = 1), otolaryngology (*n* = 1), and ophthalmology (*n* = 2), were aggregated into a composite “Other “category (*n* = 5) due to sample size constraints that precluded independent statistical analysis or reliable subgroup comparisons. Furthermore, the awarded compensation ratio was calculated by dividing the compensation awarded for the insurance dispute by the requested compensation amount and multiplying the result by 100. Cases were classified as rejected when no compensation was awarded (0%), partially compensated when the ratio was greater than 0% but less than 100%, and fully compensated when the awarded compensation equaled the requested amount (100%). In one case, the total awarded amount exceeded the requested amount because an additional SAR 43,000 was awarded separately for litigation expenses. These expenses were excluded from the compensation-ratio calculation; therefore, the case was classified as fully compensated rather than overcompensated.

#### 2.3.1 Inclusion and exclusion criteria

Judicial precedents were included if they were related to health insurance disputes in SA and officially published by the General Secretariat. Judicial precedents that were not related to health insurance disputes or were not officially published by the responsible authority were considered outside the scope of the study. No eligible cases were excluded, as all available judicial precedents contained the necessary information required for data extraction and analysis.

### 2.4 Data analysis

The primary unit of analysis for the study was the published judicial precedent. The analytical timeframe (2015–2024) covered the complete longitudinal span of all insurance judicial precedents officially published by the General Secretariat from its inception through 2024.

Data were analyzed using IBM SPSS Statistics, version 22. Descriptive statistical analysis was used to describe the data. Specifically, categorical variables were summarized using frequencies and percentages, while continuous variables were summarized using the mean, standard deviation, median, interquartile range, and range. Associations between dependent and independent variables were also examined using Fisher’s Exact test. Data not reported in the published precedents was coded as “NA.” NA values were retained as a separate category when applicable; therefore, no cases were excluded because of missing information. All cross-tabulations were based on the full sample of 38 judicial precedents (*n* = 38). A *p*-value of <0.05 was considered statistically significant.

## 3 Results

A total of 38 health insurance judicial precedents were published between 2015 and 2024. Table 2 reports the annual distribution, based on the year of the first-instance committee hearing. The year 2019 had the highest number of precedents with 15/38 (39.5%).

**Table 2:** Annual distribution of analyzed health insurance judicial precedents.

Year	Frequency of judicial precedents, *n* (%)
2015	1 (2.6%)
2016	1 (2.6%)
2017	2 (5.3%)
2018	6 (15.8%)
2019	15 (39.5%)
2020	5 (13.2%)
2021	4 (10.5%)
2022	1 (2.6%)
2023	1 (2.6%)
2024	1 (2.6%)
Not reported	1 (2.6%)

The yearly distribution is based on the first-instance committee hearing year. It reports the chronological distribution of the 38 published precedents and does not represent all cases or insurance claims that occurred in SA during those years.

As shown in Table 3, the majority of the disputes originated from the Western Region, with 16/38 cases (42.1%), while the lowest number of cases was recorded in the Eastern Region (*n* = 3/38, 7.9%). Among different medical specialties, surgery and obstetrics and gynecology were the most prevalent, each recording nine cases (23.7%). In contrast, disputes within the clinical specialty of dentistry were the least frequent accounting for four (10.5%) cases. The majority of the disputes were filed by patients (insured; 55.3%), while the lowest number of cases were filled by insurance companies with two (5.3%) cases. Most of the disputes were filed by male (*n* = 22, 57.9%) and Saudi (*n* = 17, 44.7%) plaintiffs. The results also showed that insurance companies were the defendant party in most of the disputes, accounting for 94.7% of the cases. Regarding the type of insurance coverage, medical insurance policies accounted for 65.8% of the cases, while medical malpractice policies accounted for 34.2% of the cases. The most common disputed complaint was related to the lack of insurance coverage (55.3%), while the least was insurance premium disputes (5.3%). All disputes were resolved within one year or less (94.7%), except for two cases that were resolved within 2 years.

**Table 3:** Descriptive characteristics of judicial precedent in health insurance in SA (2015–2024).

Variables	Frequency (*n* = 38)	Percentage (%)	CI 95%
Location			
Western region	16	42.1%	27.9–57.8%
Central region	8	21.1%	11.1–36.3%
Southern region	5	13.2%	5.8–27.3%
Eastern region	3	7.9%	2.7–20.8%
NA	6	15.8%	7.4–30.4%
Clinical specialty			
Surgery	9	23.7%	13–39.2%
Obstetrics and gynecology	9	23.7%	13–39.2%
Internal medicine	6	15.8%	7.4–30.4%
Dentistry	4	10.5%	4.2–24.1%
Other^1^	4	10.5%	4.2–24.1%
NA	6	15.8%	7.4–30.4%
Plaintiffs			
Patient (insured)	21	55.3%	39.7–69.9%
Healthcare provider	11	28.9%	17.0–44.8%
Other^2^	4	10.5%	4.2%–24.1
Insurance company	2	5.3%	1.5–17.3%
Gender of plaintiffs			
Male	22	57.9%	42.2%–72.1
Female	11	28.9%	17.0–44.8%
NA	5	13.2%	5.8–27.3%
Nationality of plaintiffs			
Saudi	17	44.7%	30.1–60.3%
Non-Saudi	15	39.5%	25.6–55.3%
NA	6	15.8%	7.4–30.4%
Defendant			
Insurance company^3^	36	94.7%	82.7–98.5%
Policyholder (insured)	2	5.3%	1.5–17.3%
Duration of the case			
≤1 year	36	94.7%	82.7–98.5%
>1 year	2	5.3%	1.5–17.3%
Complaint type			
Lack of insurance coverage	21	55.3%	39.7–69.9%
Violations of insurance policy	7	18.4%	9.2–33.4%
Compensation-related disputes	5	13.2%	5.8–27.3%
Public policy issues	3	7.9%	1.5–17.3%
Insurance premiums	2	5.3%	
Precedent classification			
Medical insurance policy	25	65.8%	49.9–78.8%
Medical malpractice policy	13	34.2%	21.2–50.1%
Primary verdict			
Defendant liable	29	76.3%	60.8–87%
Defendant not liable	9	23.7%	13–39.2%
Appeal committee decision			
Affirmed	29	76.3%	60.8–87%
Reversed	9	23.7%	13–39.2%
Final verdict			
Defendant liable	26	68.4%	52.5–80.9%
Defendant not liable	12	31.6%	19.1–47.5%
Ratio compensation			
Fully (100%)^4^	15	39.5%	25.6–55.3%
Rejected	12	31.6%	19.1–47.5%
Partially compensated	11	28.9%	17–44.8%

Compensation amount (SAR)	Mean ± SD	Median	IQR (Q1–Q3)	Range (Min–Max)
Requested amount (*n* = 38)	164,893.8 ± 300,992.9	57,687.8	129,386.8 (16,681.3–146,068)	1,500–1,702,500
Awarded amount (*n* = 38)	57,917.2 ± 104,660.1	16,787.8	48,000 (0–48,000)	0–500,000
Awarded amount—positive awards only (*n* = 26)	84,648.2 ± 117,721.3	42,868.5	53,982.9 (16,681.3–70,664.1)	1,500–500,000

^1^Other clinical specialties included speech therapy and occupational therapy (*n* = 1), otolaryngology (*n* = 1), and ophthalmology (*n* = 2), ^2^Other plaintiffs included healthcare entities and employers,^3^ Insurance companies include insurance companies and one third-party administrators (TPAs), ^4^ Fully compensated (100%) included one case with an additional award of SAR 43,000 for litigation expenses. This amount was excluded from the calculation of the awarded compensation ratio. NA = Not available.

In the litigation process, the first-instance committee found most defendants liable (*n* = 29, 76.3%), while nine (23.7%) defendants were found not liable. When these disputes were brought before the appeal committee, most verdicts issued by the first-instance committee were affirmed (*n* = 29, 76.3%), while nine (23.7%) verdicts were reversed. Thus, some defendants’ liability verdicts issued by the first-instance committee were changed, resulting in final verdicts of 26 liable defendants (68.4%) and 12 not liable (31.6%). The amount of compensation requested by plaintiffs ranged from 1,500 to 1,702,500 Saudi Riyals (SAR); the mean was SAR 164,893.8, (SD = 300,992.9). The amount of compensation awarded ranged from 0 SAR (rejected compensation) to SAR 500,000, with a mean of SAR 57,917.2 (SD = 104,660.1). Full compensation was awarded in 15/38 cases (39.5%). Compensation was rejected in 12/38 cases (31.6%) and partially awarded in 11/38 cases (28.9%).

The Fisher’s Exact Test was conducted to examine the association between each of the final verdict and compensation ratio with plaintiff category, precedent classification, and complaint type, as shown in Table 4. Among the 21 cases brought by an insured patient, the defendant was found liable in 14 cases (66.7%). When the healthcare provider was the plaintiff (*n* = 11), the defendant was liable in 81.8% (*n* = 9) of the cases. However, these differences in final verdicts across plaintiff categories were not statistically significant, *p* = 0.35. Additionally, Fisher’s exact test revealed no statistically significant association between the precedent classification and the final verdict of the cases (*p* = 1.00). For cases classified under a medical insurance policy (*n* = 25), the defendant was found liable in 68% of the cases. Similarly, for cases falling under a medical malpractice policy (*n* = 13), the defendant was held liable in 69.2% of the cases. Regarding complaint types, all the precedents with a complaint related to violation of insurance policy (*n* = 7) and insurance premiums (*n* = 2) were deemed liable. However, the association between the complaint type and final verdict was not statistically significant (*p* = 0.12).

**Table 4:** The association between litigation-related variables and independent variables (plaintiff, precedent classification, complaint type).

Litigation-related variables	Final verdict			Ratio compensation			
	Defendant liable (*n* = 26, 68.4%) (*n*, %)	Defendant not liable (*n* = 12, 31.6%) (*n*, %)	*p*-value	Fully (*n* = 15, 39.5%) (*n*, %)	Rejected (*n* = 12, 31.6%) (*n*, %)	Partially (*n* = 11, 28.9%) (*n*, %)	*p*-value
Plaintiff			0.350				0.230
Patient (insured)	14 (66.7%)	7 (33.3%)		7 (33.3%)	6 (28.6%)	8 (38.1%)	
Healthcare provider	9 (81.8%)	2 (18.2%)		6 (54.5%)	2 (18.2%)	3 (27.3%)	
Other	3 (50%)	3 (50%)		2 (33.3%)	4 (66.7%)	0	
Precedent classification			1.000				0.510
Medical insurance Policy	17 (68%)	8 (32%)		8 (32%)	9 (36%)	8 (32%)	
Medical malpractice policy	9 (69.2%)	4 (30.8%)		7 (53.8%)	3 (23.1%)	3 (23.1%)	
Complaint type			0.120				0.240
Lack of insurance coverage	12 (57.1%)	9 (42.9%)		6 (28.6%)	9 (42.9%)	6 (28.6%)	
Violations of insurance policy	7 (100%)	0		4 (57.1%)	0	3 (42.9%)	
Compensation-related disputes	4 (80%)	1 (20%)		2 (40%)	1 (20%)	2 (40%)	
Public policy issues	1 (33.3%)	2 (66.7%)		1 (33.3%)	2 (66.7%)	0	
Insurance premiums	2 (100%)	0		2 (100%)	0	0	

In examining the association between the compensation ratio and the plaintiff category, precedent classification, and complaint type, the results revealed no statistically significant association between any of these variables (Table 4). Specifically, the compensation ratio did not vary significantly by plaintiff category (*p* = 0.23), precedent classification (*p* = 0.51), or the complaint type (*p* = 0.24). Full compensation was awarded in 6 of the 11 cases filed by healthcare providers (54.5%) and in 7 of the 13 cases involving medical malpractice policies (53.8%). Among the seven cases involving violations of the insurance policy, four (57.1%) were fully compensated and three (42.9%) were partially compensated. Both cases involving insurance-premium complaints were fully compensated.

The Fisher’s Exact Test was conducted to examine the relationship between precedent classification with plaintiffs, complaint type, clinical specialty, final verdict, and compensation ratio, as demonstrated in Table 5. Among the 21 cases filed by an insured patient, 20 (95.2%) were classified under a medical insurance policy, and one patient was under medical malpractice. All cases were filed by healthcare providers (*n* = 11) in a medical malpractice policy case. Among complaints related to lack of insurance coverage (*n* = 21), 76.2% were classified under medical insurance policy. Fisher’s exact test revealed a statistically significant association between the precedent classification and both the plaintiff category (*p* < 0.001) and complaint type (*p* = 0.004).

**Table 5:** The association between precedent classification and different variables (plaintiff, complaint type, clinical specialty, final verdict, ratio compensation).

Litigation-related variables	Precedent classification		*p*-value
	Medical insurance policy (*n* = 25, 65.8%) (*n*, %)	Medical malpractice (*n* = 13, 34.2%) (*n*, %)	
Plaintiffs			
Patient (insured)	20 (95.2%)	1 (4.8%)	<0.001
Healthcare provider	0	11 (100%)	
Insurance company	2 (100%)	0	
Other	3 (75%)	1 (25%)	
Complaint type			
Lack of insurance coverage	16 (76.2%)	5 (23.8%)	0.004
Violations of insurance policy	1 (14.3%)	6 (85.7%)	
Compensation-related disputes	5 (100%)	0	
Public policy issues	1 (33.3%)	2 (66.7%)	
Insurance premiums	2 (100%)	0	
Clinical specialty			
Surgery	6 (66.7%)	3 (33.3%)	0.160
Obstetrics and gynecology	3 (33.3%)	6 (66.7%)	
Internal medicine	5 (83.3%)	1 (16.7%)	
Dentistry	4 (100%)	0 (0%)	
Other	2 (50%)	2 (50%)	
NA	5 (83.3%)	1 (16.7%)	
Final verdict			
Defendant liable	17 (65.4%)	9 (34.6%)	1.000
Defendant not liable	8 (66.7%)	4 (33.3%)	
Ratio compensation			
Fully (100%)	8 (53.3%)	7 (46.7%)	0.510
Rejected	9 (75%)	3 (25%)	
Partially compensated	8 (72.7%)	3 (27.3%)	

## 4 Discussion

The study analyzed health insurance judicial precedents in SA. The largest volume of these precedents originated from the Western region. Patients were the primary plaintiffs, while insurance companies predominated as defendants. The majority of the disputes were related to medical health insurance, driven primarily by complaints regarding lack of insurance coverage. The results indicated an efficient litigation process with most cases resolved within one year or less. The appeal committee affirmed most verdicts issued by first-instance committees. A total of 26 defendants were found liable and 39.5% of plaintiffs were awarded full compensation.

The study found that the majority of the disputes were filed by patients (55.3%), related to medical insurance policies (65.8%), with a main compliant related to lack of insurance coverage (55.3%). Such findings confirm with extant literature in which the majority of claim rejections were due to no coverage (7, 9, 10). These findings can be explained in light of the rapid changes that the health insurance sector in SA is witnessing. The expansion of health insurance in SA is accompanied by the emergence of new practices and cases that were not common previously, resulting in judicial precedents. Examples include disputes related to insurance coverage limits, exclusions criteria, and eligibility for insurance benefits. Furthermore, the patient population has evolved into an active group of stakeholders, represented by increased awareness of their rights and willingness to file complaints if not satisfied. The CHI received 53,000 complaints during 2017; representing an increase of up to 907% compared to 5,283 complaints recorded in 2016 (39). High rates of complaints suggest that some patients tend to file complaints even in cases that may not include an actual violation, but rather due to dissatisfaction or misunderstanding. Indeed, evidence from previous studies confirm limited or moderate knowledge of insured insurance literacy specifically related to coverage limits, exclusions criteria, network restriction, and premium ceiling. Such limited knowledge indicates a discrepancy between patient expectations and what they actually receive based on their insurance coverage (28, 40). Indeed, Alkhamis (40) explained that many insured individuals believed they understood the health insurance benefits they had. However, their actual knowledge regarding important details such as copayments and coverage limits was limited, and many participants also overestimated their understanding of insurance coverage. Consequently, formal litigation emerges not merely as a legal mechanism, but as a direct behavior of patients’ dissatisfaction stemming from limited knowledge and awareness with insurance policies.

The results found that most disputes (94.7%) were resolved within a year or less. This timely resolution indicates the speed of issuing verdicts and the efficiency of the committees. In comparison, an examination of medical malpractice cases from an insurance company in Belgium found that trial judgments required an average of 1,330 days and some took up to 2,787 days (41). These findings align with previous studies emphasizing the importance of reducing the duration of litigation procedures to increase the efficiency of judicial systems. Delaying dispute resolutions beyond a year is considered a negative indicator of the stability and efficiency of regulatory procedures (42, 43). The efficiency of the committees procedures and timely resolution can be explained by the General Secretariat’s efforts in digital transformation, as it prioritizes technology as one of its strategic and enabling programs (44). Institutional digitization efforts in insurance dispute processing were initially launched in 2016 by adopting an electronic system to manage disputes including accelerated tracks and electronic session scheduling, which directly reduce litigation periods (45).

High conformation rates at appeal levels reflect an alignment and consistency in judicial decision-making between the primary and final verdict stages, despite the disputes being settled within a short period. Furthermore, the high appellate affirmation rate observed in the study aligns with empirical baselines in general administrative and civil appellate litigation, where affirmation rates routinely exceed 70%. High affirmation rates are typically interpreted as indicators of judicial consistency, procedural stability, and mature legal standards (46, 47). However, the high affirmation rate in the findings should not be interpreted as an indicator of the performance of the committee, but rather as a measure of legal consistency and procedural concordance within published disputes. Accordingly, these precedent disputes are not attributed to the erroneous judicial operation of the committees and their processes as interpretation is confirmed across committee levels. Instead, it can be attributed to the Cooperative Health Insurance Law itself and the practice of insurers. Thus, policymakers are to benefit from these precedents and utilize them to clarify any law ambiguities, standardize any requirements, add administrative conditions as necessary to avoid or reduce the possibility of escalation to form disputes.

While clinical specialty was not directly tested for association with final verdict or compensation ratio, surgery and obstetrics and gynecology accounted for the largest proportion of total dispute cases in our descriptive analysis, each accounting for 23.7% of the cases. Existing literature documents high prevalence of litigation in these two clinical specialties (48). The nature of these clinical specialties requires urgent and rapid decisions. This may expose healthcare providers to high pressure due to the high risk, which may ultimately lead to serious complications such as delayed recovery or death (48). Thus, the concentration of disputes within surgery and obstetrics and gynecology reflects a complex intersection of coverage conflicts and outcome-related malpractice liabilities, both amplified because these specialties are expensive, procedure-heavy, and heavily judged through medical necessity lenses rather than simple diagnostic rules (49–51). Furthermore, these specialties are subject to strict insurance utilization reviews, such as prior authorizations, which frequently result in initial denials over documentation and subjective interpretations of medical necessity (49). Simultaneously, from a medical malpractice side, both fields are among the highest in medical compensation (48, 52–54). When liability is high, insurer scrutiny increases in the form of heightened documentation requirements from insurance companies.

The observed prevalence of disputes within obstetrics and gynecology and surgery carries significant implications, shifting the interpretation of health insurance disputes from isolated legal matters to indicators of broader public health and health system structural dynamic. These descriptive patterns generate important hypotheses for future empirical research. Specifically, whether recurring coverage denials and malpractice claims in high-intensity specialties actively contribute to provider burnout, defensive clinical practices, compromised continuity of care, or system-wide cost inflation remains a critical subject for prospective research. Prior literature establishes that when certain clinical specialties dominate litigation, physicians practice defensive medicine. Such practice can delay clinical decisions, increase burnout, raise costs, and risk an effective patient-physician relationship, ultimately compromising patients’ quality and continuity of care (55, 56).

The study’s findings were based on the analysis of 38 appealed and published judicial precedents, however some methodological and structural limitations should be considered when interpreting the findings. First, judicial precedents are selectively published by the General Secretariat and do not represent an exhaustive record of all filed health insurance disputes in SA. Thus, published precedents represent a selective corpus of fully adjudicated and appealed disputes; omitting unresolved, pending, or settled disputes and thus lack of representativeness of all disputes. Second, the sample size (*n* = 38) constrains statistical power and limits the granularity of actionable policy recommendations. Specifically, the non-significant results across all six Fisher’s exact tests are very plausibly attributable to low statistical power rather than an absence of underlying association between case characteristics and judicial outcomes. Consequently, the non-significant findings are interpreted cautiously as inconclusive. Future research utilizing larger, longitudinal data as judicial transparency expands will be essential to achieve adequate power for sub-group analyses to reach solid conclusions. Third, because the published precedents do not follow a standardized reporting format, the level of details within the records was occasionally inconsistent, as some cases missed specific information and did not allow for data extraction. Finally, evaluating true operational efficiency of committees requires further considerations such as comparative benchmarks, case-complexity adjustments, and tracking of pending cases, which were unavailable for this dataset. Consequently, the study findings can be utilized as an empirical baseline at a moment of substantial regulatory transition in SA and offers a useful benchmark for future longitudinal comparisons.

The study’s findings highlight areas for future research. Longitudinal studies with larger sample sizes will be necessary to track evolving litigation trends and confirm whether the high appellate affirmation rates observed in this study remain stable over time. Additionally, future investigations should employ qualitative or mixed-methods approaches, such as conducting structured interviews with insurance compliance officers and judicial committee members, to uncover the specific operational and institutional factors driving the high concentration of disputes within certain clinical specialties. Finally, researchers should pair judicial dataset analyses with empirical surveys measuring patient health insurance literacy and satisfaction post-litigation. This would allow researchers to understand how levels of satisfaction and insurance literacy reflect validity of filed disputes.

The findings of the study highlight several implications for policymakers and healthcare practitioners as SA’s health insurance sector continues to expand. Key priorities include enhancing the transparency and systematic publication of judicial precedents and standardizing the formal documentation of claim denial reasons. Additionally, the analysis of the unique judicial precents from the appeal committee is imperative as it contributes to refining existing laws and regulations. Such analysis and results provide insights to lawmakers and policymakers on the efficiency of the existing laws and regulations, highlighting aspects of laws where revision or expansion is needed to clarify and increase efficiency of relevant processes. Furthermore, it is essential to enhance coordination among relevant multisectoral regulatory bodies, such as the Insurance Authority, which is responsible for regulatory oversight of the insurance sector, and the Council of Health Insurance to oversee the implementation of the health insurance system. This coordination could enhance and develop unified regulations and operations to ensure efficient workflow and keep pace with the continuous changes in the health insurance sector in SA.

## 5 Conclusion

This study provides an empirical analysis of judicial precedents within Saudi Arabia’s health insurance sector demonstrating high stability and procedural conformity across committee levels. The heavy concentration of cases involving medical coverage denials and patient-led litigation underscores the critical need for clear regulatory frameworks to minimize disputes between insurers, providers, and beneficiaries. For policymakers and healthcare practitioners, key priorities include clarifying coverage and exclusion terms in policy contracts and standardizing the formal documentation of claim denial reasons to improve administrative efficiency and judicial consistency. The study’s findings lay a foundation for future longitudinal and multi-jurisdictional research as Saudi Arabia’s health sector reforms continue to evolve.

## Data Availability

Publicly available datasets were analyzed in this study. This data can be found here: https://idc.gov.sa/en-us/Pages/Library.aspx?tab=tabpane_3/.
